# Research Trend and Detailed Insights into the Molecular Mechanisms of Food Bioactive Compounds against Cancer: A Comprehensive Review with Special Emphasis on Probiotics

**DOI:** 10.3390/cancers14225482

**Published:** 2022-11-08

**Authors:** Manas Yogendra Agrawal, Shreyas Gaikwad, Sangeeta Srivastava, Sanjay K. Srivastava

**Affiliations:** 1 Department of Immunotherapeutics and Biotechnology, Texas Tech University Health Sciences Center, Abilene, TX 79601, USA; 2 Center for Tumor Immunology and Targeted Cancer Therapy, Texas Tech University Health Sciences Center, Abilene, TX 79601, USA; 3 Department of Chemistry, Lucknow University, Lucknow 226007, India

**Keywords:** cancer, bioactive, phytochemicals, cruciferous vegetables, probiotics, nanoformulation, chemotherapy, apoptosis, oncogene, chemoprevention, curcumin, ROS

## Abstract

**Simple Summary:**

Cancer is one of the leading causes of death worldwide. Treatment of cancer has long been a challenge. While researchers have been searching for many options for the cure, mother nature has blessed us with natural bioactive components with anticancer potential. Since the 1800s, scientists have been studying the efficacy of the bioactive agents present in our food for the treatment of cancer. This review summarizes the molecular mechanisms responsible for these effects. Moreover, owing to the increased intake of probiotics in daily diets, this review also explains how they can be helpful in cancer prevention and treatment.

**Abstract:**

In an attempt to find a potential cure for cancer, scientists have been probing the efficacy of the food we eat and its bioactive components. Over the decades, there has been an exponentially increasing trend of research correlating food and cancer. This review explains the molecular mechanisms by which bioactive food components exhibit anticancer effects in several cancer models. These bioactive compounds are mainly plant based or microbiome based. While plants remain the primary source of these phytochemicals, little is known about probiotics, i.e., microbiome sources, and their relationships with cancer. Thus, the molecular mechanisms underlying the anticancer effect of probiotics are discussed in this review. The principal mode of cell death for most food bioactives is found to be apoptosis. Principal oncogenic signaling axes such as Akt/PI3K, JAK/STAT, and NF-κB seem to be modulated due to these bioactives along with certain novel targets that provide a platform for further oncogenic research. It has been observed that probiotics have an immunomodulatory effect leading to their chemopreventive actions. Various foods exhibit better efficacy as complete extracts than their individual phytochemicals, indicating an orchestrated effect of the food components. Combining bioactive agents with available chemotherapies helps synergize the anticancer action of both to overcome drug resistance. Novel techniques to deliver bioactive agents enhance their therapeutic response. Such combinations and novel approaches are also discussed in this review. Notably, most of the food components that have been studied for cancer have shown their efficacy in vivo. This bolsters the claims of these studies and, thus, provides us with hope of discovering anticancer agents in the food that we eat.

## 1. Introduction

The American Cancer Society states, “Cancer is a group of diseases characterized by uncontrolled growth and spread of abnormal cells” [1]. The multifaceted adverse effects of cancer are the reasons for its mention as a “group of diseases”, as it leads to several physiological adversities, which, if not treated, lead to death. Humans have been fighting a long-lasting battle with this disease with few positive results, even though cancer has been studied extensively by researchers. Cancer research encompasses (1) the study of the mechanisms by which it initiates and progresses, and (2) the study of the mechanisms by which anti-cancer agents act. Successful and in-depth studies of these aspects bolster the foundation for further discoveries of potential anti-cancer agents. Scientists have not been able to find any absolute cure for cancer. Several direct and indirect approaches have been attempted to treat cancer. The use of antioxidants [2] and anti-inflammatory agents [3] reduce the tumor-friendly environment and thereby alleviate cancer. Repurposing the existing FDA-approved non-cancer drugs to treat cancer is another promising strategy and has been studied rigorously [4,5,6,7]. This approach could reduce the long wait times and avoid the millions of dollars needed for new drug development. Strategies such as radiotherapy and surgery are used to eliminate the majority of the tumor. The remainder of the tumor can further be treated using chemotherapies.

**Figure 1:**
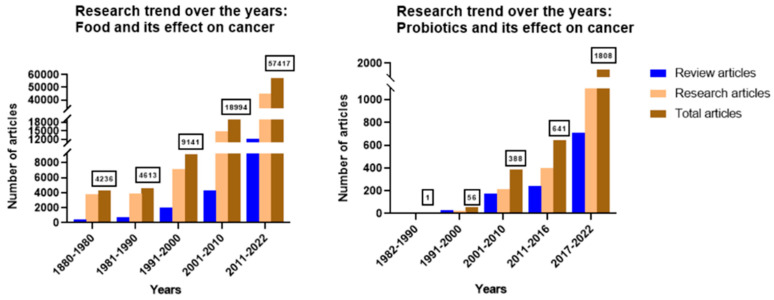
Research trend of food and its association with cancer over the years (**left**); research trend of probiotics and cancer over the years (**right**). The data related to the number of publications was curated from the PubMed database (https://pubmed.ncbi.nlm.nih.gov/, accessed on 1 June 2022). (Figure created with Biorender.com (https://biorender.com/, accessed on 1 June 2022) and GraphPad Prism, version 9.4.1 (GraphPad Software, San Diego, CA, USA)).

In an effort to discover agents with anti-cancer efficacy, bioactive compounds found in nature have been studied extensively. Moreover, regular vegetables have phytochemicals that exhibit anti-neoplastic properties [8]. As the available chemotherapies exhibit significantly high toxicity, there is a good rationale to identify non-toxic chemicals from fruits and vegetables for the treatment of cancer. As seen in Figure 1, over the years, researchers have been studying the association between food and cancer. The number of scientific publications in the decade from 1980 to 1990 was equal to the total number of publications in the century from 1880 to 1980. These publications are only increasing with time. The number of review articles has been increasing exponentially with time, signifying a growing interest among researchers in deciphering the anticancer potential of food bioactives. Probiotics have become an essential part of the modern daily diet. Their potential against cancer has been studied since 1982 and has been at its peak since the last decade (Figure 1). Thus, this review not only focuses on plant-based food bioactives but also probes the molecular mechanisms by which probiotics help combat cancer.

It has been observed in several studies that the food matrix as a whole, rather than the individual phytochemicals, exerts synergistic effects. This is because of the orchestrated effect of the entire food matrix compared with the physiological effects of chemotherapies and drugs. This narrative review showcases several of such examples. Although there is a huge variety of foods with anti-cancer potential, those covered in this review have been studied both in vitro and in vivo. A summary of the list of functional foods covered in this review is provided in Appendix A.

## 2. Plant-Based Food Components

Plants serve as the major source of food for living beings and thus have been studied the most for their anti-cancer potential. Plants have evolved over centuries to produce secondary metabolites such as flavonoids, terpenoids, and alkaloids with diverse functions. Plant-based foods belonging to different categories and their active phytochemicals are discussed below.

### 2.1. Peppers and Other Nightshade Vegetables

Different kinds of peppers, tomatoes, and eggplant fall under the category of nightshade vegetables belonging to the Solanaceae family. Peppers are an essential part of almost all cuisines around the world. Several phytochemicals that exert the pungent and hot taste in peppers have also been found to exhibit anti-cancer effects in several cancer models. Eggplant and tomatoes have also shown anticancer effects in different experimental models. The molecular mechanisms involved in the anti-cancer effects of nightshade vegetables are elaborated below.

#### 2.1.1. Black Pepper (*Piper nigrum*)

Piperine and piperidine are the alkaloids in black pepper, making it pungent. Both alkaloids show a strong anti-cancer effect, as discussed below.

##### Piperine

Piperine exhibits a wide range of physiological activities such as anti-depressant, anti-arthritic, and anti-inflammatory effects, along with neurological activities such as mood elevation and treatment of cognitive disorders [9,10]. Due to the promising physiological effects of this alkaloid, piperine has also been studied for cancer. It is effective against melanoma [11], breast cancer [12], ovarian cancer [13], gastric cancer [14], glioblastoma [15,16], lung cancer [17], oral squamous carcinoma [18,19,20], prostate cancer [21,22,23,24], rectal cancer [25,26,27], cervical cancer [28], pancreatic cancer, and leukemia [29].

PI3K/Akt signaling has been recognized as the clinical target for breast cancer treatment [30]. Piperine decreased the phosphorylation of Akt at the Ser473 residue, leading to apoptosis [31]. Also, it was able to reduce the mitochondrial membrane integrity with the release of mitochondrial cytochrome c and Smac/DIABLO into the cytosol. Cytochrome c in the cytosol forms apoptosome activating caspase-9, the initiator caspase [32]. Smac/DIABLO inhibits the inhibitors of apoptosis (IAP) proteins, which are responsible for the inhibition of caspase activity, thereby making apoptosis-mediated cell-death even stronger [33]. MMP-2 and MMP-9, responsible for tumor metastasis, were inhibited by piperine treatment as well. Piperine also inhibits signal transducer and activator of transcription 3 (STAT3), p65, and IκBα, leading to the downregulation of Bcl-2 and thus causing apoptosis [31].

Si et al. studied the molecular mechanism underlying the antiproliferative effects of piperine on ovarian cancer cells (A2780). They confirmed apoptosis as the mode of cell death by confirming the increase in cytochrome c levels. Cytochrome c is released by mitochondria due to damage to the mitochondrial membrane potential. Further, they observed an increase in caspase-3 and caspase-9 levels, along with an increase in the expression of cleaved PARP, confirming the activation of the intrinsic apoptotic machinery. Unchanged levels of caspase-8, which plays a role in activating the extrinsic apoptotic pathway [34], indicated that cell death occurred via the intrinsic apoptosis pathway. JNK and p38 MAPK signaling axes were also modulated due to piperine, thus proposing the inhibition of JNK and p38 MAPK to be the probable mechanism of action [13].

It is known that IL6 is responsible for gastric cancer induction and leads to cancer cell invasion by activating the c-Src/RhoA/ROCK axis. STAT3 activation also leads to an increase in the production of IL-6. Apart from STAT3, IL-1ß also induces IL-6 production through PI3 K-dependent Akt/IκB signaling. In gastric cancer cells, IL-1ß activates the major MAPK pathways, *viz*, p38 MAPK, ERK 1/2, and JNK/SAPK [35,36,37,38]. The antiproliferative effect of piperine against gastric cancer cells (TMK-1) was found to be due to inhibition of IL-6, along with the inhibition of STAT3 and p38 MAPK pathways [14].

Piperine was found to increase caspase-3, -8, and -9 expressions, resulting in the induction of apoptosis in glioblastoma (GBM) cells. Piperine also inhibits the expression of CDK2-cyclin-E and CDK-4/6-cyclin D complexes, suggesting a G1/S cell-cycle arrest. It also increases the phosphorylation of the JNK/p38 MAPK signaling pathway, thereby curbing glioma development [16]. Thus, piperine inhibits glioblastoma tumor growth by modulating the JNK/p38 signaling axis.

Lin et al. found an increase in the expression of tumor suppressor p53 gene by piperine treatment in human lung cancer cells (A549). As p53 is responsible for inducing G2/M cell-cycle arrest [39,40], the authors proposed that piperine might possibly cause G2/M cell-cycle arrest in lung cancer cells. Increased caspase-3 and -9 activity, along with increased Bax and decreased Bcl-2, confirmed apoptosis as the cell death mode. Unchanged caspase-8 confirmed the apoptotic pathway to be intrinsic [17]. Thus, piperine alleviates lung cancer by increasing p53 expression and arresting them in the G2/M phase along with the intrinsic apoptosis of cells.

Researchers have shown that piperine inhibits the proliferation of leukemic cells (HL60 and K-562), inducing both extrinsic and intrinsic apoptosis [29,41]. Piperine increased the expression of caspase-3, -8, and -9 with an increase in Bax and a decrease in Bcl-2.

Piperine modulates STAT-3 and NF-κB signaling in cervical cancer cells [42]. Inhibition of rectal cancer by piperine can be attributed to the modulation of Wnt/catenin signaling along with an increase in ROS production [43]. Piperine has also been studied against pancreatic, oral squamous, and prostate cancers.

##### Piperidine

Piperidine is another strong alkaloid in black pepper that has been studied broadly for its anti-cancer potential. A study showed the antiproliferative effects of piperidine against both estrogen receptor-negative and -positive MDA-MB-231 and MCF-7 cells, respectively. It restricted the cell cycle in the G0/G1 phase [44]. Piperidine also inhibited prostate cancer cell growth by the induction of apoptosis [45]. It increased the levels of pro-apoptotic Bax and decreased the expression of BcL-2 and XIAP [24]. Epithelial–mesenchymal transition (EMT), a regulatory step in prostate cancer, is activated during the migration of cancer. A piperidine derivative, 17a, has been shown to hinder the cell migration of prostate cancer cells. E-cadherin, a marker of epithelial cells, was seen to be upregulated, whereas N-cadherin and vimentin, markers of mesenchymal cells, were downregulated. Moreover, 17a inhibited tubulin polymerization by binding to colchicine binding sites [24]. The piperidine derivative 2-amino-4-(1-piperidine) pyridine has shown anti-proliferative effects against HT29 and DLD-1 colon cancer cells, with G0/G1 cell cycle phase arrest [46,47]. It downregulated FOXA2 mRNA, responsible for the proliferation and metastasis of colon cancer cells. Moreover, the expression of mesenchymal cell marker vimentin decreased, suggesting a decrease in the epithelial–mesenchymal transition (EMT) by the piperidine derivative treatment [47]. Thus, piperidine has exhibited significant anti-cancer activity against breast, prostate, colon, lung, and ovarian cancers.

#### 2.1.2. Long Pepper (*Piper longum*)

Long pepper is a spice that hails from India. The word pepper derives from the Sanskrit name *pippali*. Later, this spice spread to Greece, followed by Rome and greater Europe. Today, long pepper is used worldwide. Long pepper has been shown to be effective against renal cancer. Piperlongumine (PL), also known as piplartine, is the principal alkaloid present in long pepper. It acts through the downregulation of c-Met protein. Hepatocyte growth factor (HGF) is the ligand for c-Met and is responsible for cancer cell proliferation, growth, motility, and migration. Piperlongumine exerts its effect via the ROS-dependent mechanism. Moreover, the c-Met depletion by PL coincides with the downregulation of downstream signaling pathways such as STAT3, NF-κB, PI3K/Akt, and ERK/MAPK. The study also discovered that the PL derivatives, PL-fluorophenyl (PL-FPh) and PL-dimer (PL-Di), exhibited much better efficacy than PL alone. The subcutaneous xenograft model of the PNX0010 cells showed inhibition of tumor growth by PL treatment, which increased with PL-Di. Thus, these findings strengthen the claim of the efficacy of long pepper against renal cell carcinoma [48]. In a study by Conde et al., PL inhibited the glioblastoma tumor progression in vivo in the orthotopic models of U87- and U251-injected mice. There was also a decrease in malignant cells derived from the patient’s primary tumors. The study found that hTRPV2 was responsible for the sensitivity of PL’s effect. Knockdown of hTRPV2 led to decreased sensitivity of PL and the production of ROS. Thus, PL’s anti-tumor effect can be attributed to the upregulation of hTRPV2 [49]. Harshbarger et al. found GSTP1 to be another target for cancer inhibition by PL. GST is an antioxidant enzyme. It has various isozymes. Expression of GSTP1 in cancer cells correlated with resistance to chemotherapy. It was observed that PL underwent hydrolysis intracellularly to form the hydrolysis product of PL (hPL), which binds to GSH. This hPL–GSH complex binds to the GSTP1 site leading to the blockade of GSTP1 and thereby its enzymatic activity. Also, this study showed that PL acts as a prodrug to elicit its action. The hPL–GSH complex further leads to a decrease in GSH levels and an increase in ROS, leading to apoptosis. These observations were made in the cervical (HeLa), pancreatic (PANC1), and colorectal (SW620) cancer cells [50]. Altogether, piperlongumine can be considered as a promising anti-cancer bioactive agent that acts through the downregulation of c-Met, STAT3, NF-κB, PI3K/Akt, and ERK/MAPK signaling pathways, inhibition of GSTP1, and upregulation of hTRPV2.

#### 2.1.3. Chili Pepper (*Capsicum annuum*)

Chili peppers consist of an alkaloid, capsaicin, a potent physiologically active natural compound. Although capsaicin’s anti-cancer potential was controversial in the initial phase [51,52], current research has provided a solid basis to confirm that capsaicin exhibits anti-cancer potential [53,54,55,56,57,58].

Zhang et al. observed significant inhibition of the growth of pancreatic cancer cells (AsPC-1 and BxPC-3) by capsaicin treatment. This effect was attributed to the induction of apoptosis, ROS generation, and mitochondrial membrane potential disruption. Apoptosis was confirmed by the up-regulatory effect of capsaicin on Bax along with the downregulation of Bcl-2 and survivin. The release of cytochrome c and apoptosis-inducing factor (AIF) in the cytosol was also observed. Capsaicin enhanced the expression of JNK, thus suggesting that its action is through the JNK signaling axis. Inhibition of ß-catenin is also a mechanism by which capsaicin inhibits pancreatic tumor growth. The authors also confirmed these findings in vivo, wherein they observed that oral administration of capsaicin leads to the significant inhibition of AsPC-1 pancreatic tumor xenografts in athymic nude mice without any side effects [53]. Pramanik et al. delineated the mechanism of ROS generation by capsaicin. The authors found that ROS generation was associated with the inhibition of mitochondrial complex-I and complex-III by capsaicin in BxPC-3 and AsPC-1 human pancreatic cancer cells. The findings were confirmed when the authors observed no ROS generation in BxPC3-rho (ρ0) cells with a dysfunctional mitochondrial oxidative phosphorylation system. These results were also reproduced in vivo [54]. Thus, to summarize, capsaicin inhibits pancreatic tumor growth both in vitro and in vivo by the modulation of JNK signaling and ROS generation and leads to inhibition of mitochondrial complexes I and III. Capsaicin has also been effective in other cancer models such as bladder, renal, hepatic, breast, and oral carcinoma. Capsaicin’s anti-cancer effect has been observed through the binding to transient receptor potential cation channel subfamily V member 1 (TRPV1), which leads to an increase in intracellular calcium and thus apoptosis [59]. Activator protein 1 (AP-1), nuclear factor kappa B (NF-κB), and STAT3 are signaling pathways responsible for tumor growth. Studies have shown that capsaicin inhibits AP1, NF-κB, and STAT3 in cancer cells [60]. Islam et al. have also shown that capsaicin binds to sirtuin 1 (SIRT1), leading to down-regulation of SIRT1 deacetylase, which reduces the migration of bladder cancer cells [61]. Thus, chili pepper compounds exhibit significant antineoplastic effects in several cancers.

#### 2.1.4. Other Nightshade Vegetables

##### Eggplant (*Solanum melongena*)

Eggplant has been shown to exert anti-cancer effects in fibrosarcoma as well as ovarian, skin, lung, gastric adenocarcinoma, and liver cancer models [62,63,64,65,66]. Eggplant consists of glycoalkaloids such as solasodine, solasonine, and solamargine, which exhibit anti-cancer effects [67]. Downregulation of the matrix metalloproteases and miR-21 is the mechanism by which food bioactives in eggplant exhibit anti-cancer effects [65]. Although there are not many molecular studies available on the efficacy of eggplant on skin cancer, several clinical trials have been conducted, testing the efficacy of eggplant on skin cancer.

##### Tomato (*Solanum lycopersicum*)

Tomato is the second-most important fruit or vegetable after potato in the cuisine world. Tomatidine, an alkaloid present in the leaf of tomatoes, has been shown to be useful against gastric cancer via the regulation of interferon-stimulated genes (ISGa) [68]. Carotenoids present in tomatoes are strong antioxidants and thus help in preventing the damage caused to the DNA. Lycopene, a carotenoid, is another antioxidant from tomato that has been effective against prostate cancer. It enhances the sensitivity of prostate cancer to the anti-cancer drug enzalutamide by modulating the AKT/EZH2/androgen receptor-signaling pathway. The in vivo findings indicated a significant inhibition of the tumor growth by carotenoid treatment as compared to control, along with a reduction in bone metastasis [69]. It was also observed that whole tomato powder (10% in diet), which contained other carotenoids, was more effective in alleviating prostate cancer than lycopene alone (0.025%) [70]. Lycopene’s activity has also been studied in vivo. Gupta et al. observed that lycopene at a dose of 5 mg/kg was able to downregulate cyclin D1, HIF-1α, and PCNA, thus reducing hepatocellular carcinoma growth [71]. Tomatoes and their bioactive components have shown promise in suppressing lung and breast cancers as well [72,73]. There are a few studies that have observed almost no effect of tomatoes on prostate cancer [74]. However, the tomato–lycopene–prostate cancer triad has been studied extensively with promising outcomes from most of the studies [75].

Thus, solanaceous nightshade vegetables, including peppers, exhibit promising anti-cancer potential in different types of cancers. This effect is exerted via the modulation of several proto-oncogenic pathways, both in vitro and in vivo.

### 2.2. Spices

Spices have been known to exert numerous physiological functions useful against different diseases along with strong anti-cancer potential. Clove and turmeric are the spices with strong anti-cancer potential and are discussed below.

#### 2.2.1. Cloves (*Syzygium aromaticum*)

Clove oil extract has shown antiproliferative effects in breast cancer cells (MCF-7 and MDA-MB231), cervical cancer cells (HeLa), prostate cancer cell metastasis of the brain (DU145), and esophageal cancer cells (TE-13) [76,77]. *Helicobacter pylori* (HP), a Gram-negative bacterium, is known to cause gastric cancer. The methanolic extract from the leaves of cloves inhibits the growth of all the strains of HP. The authors extrapolated these finding to the Thai population that is less susceptible to gastric cancer despite having a higher incidence of HP infections, as the Thai cuisine abundantly uses cloves [78]. This shows that the use of cloves can serve as a preventive measure for gastric cancer by inhibiting HP growth. Liu et al. found that oleanolic acid (OA), a phytoconstituent present in cloves, exhibit anti-cancer properties. However, a comparative study found that the whole extract of cloves was more effective than the bioactive constituents alone. This indicates that natural food components exert their effect in an orchestrated fashion, where all the phytoconstituents work in synergy to achieve the desired effects. However, the identification of a particular phytochemical will be helpful for further drug development processes. The study also strengthened the claim by showing that an ethyl acetate cloves extract at a 50 mg/kg dose exerted maximum inhibition of subcutaneous colorectal adenocarcinoma tumors (HT29) in mice. The effect was better than the individual treatments of oleanolic acid and standard therapy 5-fluorouracil. The cloves extract arrested the cell growth in the G0/G1 phase and also exhibited an increase in apoptosis in a dose-dependent fashion. The cloves extract and OA treatment led to the downregulation of cell-cycle proteins such as E2F1 and increased the protein expression of p21 WAF1/Cip1 and γ-H2AX. Downregulation of thymidylate synthase hints towards DNA damage [79]. Kubatka et al. saw a significant inhibition in breast tumor growth in mice following cloves administration compared to the control. Enhanced caspase-3 and Bax confirmed apoptosis as the mode of cell death. Cloves treatment also increased the population of cells with depleted MMP, thus showing that cloves also acts through the mitochondrial apoptosis pathway. Ki67, a proliferation marker, and VEGFA, responsible for angiogenesis, were downregulated in mice fed with a cloves-rich diet. CD44, CD24, and ALDH1 are markers for cancer stem cells (CSC) in breast cancer. All three CSC markers were downregulated due to a cloves diet [80]. Li et al. studied the effects of aqueous extracts of cloves in pancreatic and colon cancer models. The extract induced autophagy in cancer cells. The role of the AMPK pathway in the autophagy process is well known. An aqueous extract of cloves led to an increase in AMPK and ULK, thus proposing modulation of AMPK/ULK-mediated autophagy to be the probable mechanism. Colon tumor growth in mice was inhibited by oral administration of a cloves extract. The inhibition was higher than the standard cyclophosphamide therapy [81]. Nirmala et al. developed an oil-based nanoscale emulsion of cloves buds and tested its anti-cancer efficacy against thyroid cancer cells (HTh-7). The nanoscale emulsion showed anti-proliferative effects against thyroid cancer cells, with apoptosis seen as the mode of cell death [82]. This study provides a novel method for the delivery of cloves. Thus, it can be said that the phytochemicals in cloves along with the whole cloves bud and its extract helps combat cancer.

#### 2.2.2. Turmeric (*Curcuma longa*)

Turmeric is another famous spice used in various cuisines worldwide. Historically, turmeric has been used by South Asian populations as an antiseptic in healing wounds and as an anti-inflammatory agent. The most active phytocompound in turmeric is the beta-diketone curcumin, which has been studied in-depth for its anti-cancer effects in numerous cancers. Turmeric is been tested in clinical trials for almost all types of cancers.

Curcumin, the main chemical present in turmeric, has been found by Sahu et al. to inhibit pancreatic cancer cell growth, with apoptosis being the mode of cell death. Curcumin also arrests the growth of pancreatic cancer cells (BxPC-3) in the G2/M phase. Phosphorylation of H2A.X and Chk1, the markers of DNA damage, were upregulated, whereas DNA polymerase-ß, a DNA repair enzyme, was downregulated due to curcumin treatment. In addition, ATM phosphorylation was increased due to curcumin treatment along with a decrease in cyclin B1, confirming the G2/M arrest of cells. It can be concluded that ATM/Chk1 plays an important role in mediating the G2/M arrest of cells caused by curcumin, leading to the anti-tumor effects against pancreatic cancer [83]. Curcumin also increases the sensitivity of non-small lung cancer cells to cisplatin, the standard therapy. This effect was regulated via the endoplasmic reticulum stress pathway [84]. Curcumin suppressed the Akt/PI3K/mTOR signaling axis and upregulated miR-199a to inhibit oral squamous cell carcinoma [85]. Curcumin suppressed papillary thyroid cancer by modulating the long non-coding RNA LINC00691 through the Akt signaling axis [86]. Curcumin also works on several other cancers such as glioblastoma by acting through MMP, NF-κB, STAT3, and PI3K/Ak/mTOR downregulation [87]; breast cancer by p53 regulation [88], and lung cancer metastasis inhibition by regulating the adiponectin/NF-κB/MMPs signaling pathway [89].

CLEFMA (4-[3,5-bis(2-chlorobenzylidene-4-oxo-piperidine-1-yl)-4-oxo-2-butenoic acid]), is a curcuminoid that exhibits anti-cancer properties [90]. CLEFMA has been proposed to elicit its anti-cancer effects by perturbating redox homeostasis in cancer cells [91]. It also mediates cell death via the intrinsic apoptosis pathway, leading to the activation of procaspase-3 and procaspase-9 [92]. CLEFMA also induces apoptosis by increasing BAX and BID [93]. It down-regulates pro-apoptotic proteins by acting on the NF-κB pathway [90,94,95]. Moreover, it also suppresses expression of the pro-inflammatory COX2 [90]. CLEFMA also decreased the expression of cyclin D and caused cell-cycle arrest in the S phase of H441 cells. CD31, a marker of angiogenesis, and ICAM1, which is responsible for cell migration and adhesion, were also downregulated by CLEFMA treatment [90]. Several other spices with anti-cancer properties are black cumin (*Nigella sativa*), rosemary (*Salvia rosmarinus*), saffron (*Crocus sativus*), oregano (*Origanum vulgare*), and basil (*Ocimum basilicum*) [96]. All these spices have been studied for numerous cancer models with positive results.

### 2.3. Cruciferous Vegetables

Cruciferous vegetables belong to the Brassicaceae (formerly Cruciferae) family, which include Broccoli (*Brassica oleracea var. italica*), Cabbage (*Brassica oleracea var. capitata*), Cauliflower (*Brassica oleracea var. botrytis*), Kale (*Brassica oleracea var. sabellica*), Mustard (*Brassica juncea*), Watercress (*Nasturtium officinale*), Horseradish (*Armoracia rusticana*), etc. Cruciferous vegetables have been studied extensively for their antineoplastic potential. These vegetables have a few common phytochemicals, such as isothiocyanates, diindolylmethane, and sulforaphane, which exert anti-cancer effects. Molecular mechanisms underlying the anti-carcinogenic effect of these phytocompounds are discussed below.

#### 2.3.1. Isothiocyanates (ITC)

ITCs suppress the carcinogen activation and increase the detoxification of the same. Glucosinolates store ITCs in cruciferous vegetables. Even the glucosinolates have been found to exert anti-cancer effects. Overall, ITCs exert their anti-cancer effect by inducing oxidative stress, apoptosis, and cell-cycle arrest, inhibiting the tumor’s metastasis and inhibiting angiogenesis [97]. Three main isothiocyanates that are extensively studied are benzyl isothiocyanate (BITC), phenethyl isothiocyanate (PEITC), and sulforaphane, as discussed in this review. In addition to cruciferous vegetables, BITC is also found in papaya seeds (*Carica papaya*).

##### Benzyl Isothiocyanate (BITC)

BITC has several beneficial physiological effects along with its anti-cancer potential against different cancers such as breast cancer [98], non-small lung cancer [99], prostate cancer [100], leukemia [101], colon cancer [102], hepatocellular cancer [103], and pancreatic cancer [104]. BITC also sensitizes the tumors to standard chemotherapies, thereby diminishing the problem of drug resistance [105].

BITC treatment caused DNA damage in cells, which resulted in G2/M cell-cycle arrest in pancreatic cancer cells. The mode of cell death against pancreatic cancer cells was apoptosis [106]. Sahu et al. also confirmed that apoptosis by BITC was selective to cancer cells but did not affect the viability of normal human pancreatic ductal epithelial (HPDE) cells. They found that the apoptosis induced in pancreatic cancer cells was through the inhibition of STAT3 signaling. They also showed the tumor-suppressing efficacy of BITC in BxPC3 tumor xenografts in mice [104]. In another study, inhibition of the PI3K/AKT/FOXO pathway was found to be another mechanism by which BITC exerted its effect against pancreatic cancer [107]. Boreddy et al. found the inhibition of HIF-1α/VEGF/Rho-GTPases by STAT3 to be the reason for suppression of angiogenesis and invasion of pancreatic tumors [108]. Thus, BITC is a strong anti-cancer agent effective against pancreatic cancer acting via inhibition of PI3K/AKT/FOXO-, STAT-3-, and STAT-3-mediated HIF-1α/VEGF/Rho-GTPases signaling axes. Lai et al. found the effectiveness of BITC against colon cancer, where they found that BITC was able to inhibit the migration and invasion of human colon cancer cells. This effect was due to the downregulation of urokinase-type plasminogen activator (uPA) linked to protein kinase C (PKC), the MAPK signaling pathway, and the MMP-2/9 pathway [102]. The anti-tumor effect of BITC was confirmed in breast cancer, which showed potentiation of p53 signaling. The activation of p53 was extrapolated to the activation of p53-LKB1 and p73-LKB1 axes. The mammosphere-forming ability of breast cancer cells was also diminished by BITC treatment [109]. Overall, it can be concluded that BITC exerts strong anti-cancer potential through the inhibition of several oncogenic pathways and has therapeutic selectivity towards cancer cells, averting the cytotoxic effects on normal cells.

##### Phenethyl Isothiocyanate (PEITC)

Multiple researchers have established the efficacy of PEITC, both in vitro and in vivo experimentally in various cancers such as prostate, breast, cervical, lung, colorectal, and metastatic breast cancer, as well as leukemia and glioblastoma. Gupta et al. observed that oral administration of PEITC suppresses the metastasis of breast tumor cells to the brain. This effect was observed within days of PEITC administration in an in vivo study exhibiting its high potency [110]. This research group established HER2 as the potential target of PEITC in breast cancer. PEITC also enhanced the sensitivity of breast tumors towards the standard therapy, such as doxorubicin, exhibiting synergistic effects [111]. PEITC was also shown to induce immune modulation in tumor-bearing mice xenografts. The suppression of breast tumor growth was associated with the reduction in myeloid-derived tumor suppressor cells (MDSCs) and T regulatory lymphocytes [112]. Boyanapalli et al. showed that PEITC treatment resulted in the arrest of prostate cancer cells in the G2/M phase and the induction of apoptosis. This effect of PEITC was due to the reactivation of the tumor suppressor RASSF1A [113]. Wu et al. observed a significant reduction in the tumor incidence in the transgenic TRAMP model of prostate cancer in mice fed with PEITC supplemented diet. The effect was attributed to the attenuation of cell-cycle/Cdc42 signaling in PEITC-fed mice and an inverse regulatory relationship between the RNA expression of Adgrb1 and Ebf4 genes and CpG methylation [114]. Thus, it can be said that PEITC attenuates prostate cancer growth by the activation of RASSF1A, cell-cycle arrest, and reduced inflammation along with an impact on the global CpG epigenome and transcriptome. PEITC was also effective against leukemia. Liu et al. found that PEITC induced ROS generation in chronic lymphocytic leukemia (CLL) cells deficient in p53. Similar results were also observed in vivo, where PEITC significantly increased the survival of p53 knock-out mice as compared to the control group [115]. PEITC could likely be effective against CLL, with patients having p53 mutations. It is known that several metabolic alterations occur in the immune cells due to cigarette smoke [116,117,118,119,120]. Yuan et al. observed that PEITC reduced the metabolic activation of 4-(methylnitrosamino)-1-(3-pyridyl)-1-butanone (NNK), a tobacco carcinogen, by 7.7% in smokers [121]. Taken together, PEITC is a promising phytochemical against several cancers.

##### Sulforaphane (SFN)

Sulforaphane, an isothiocyanate, is a well-known phytochemical present in broccoli and other cruciferous vegetables. It has exhibited its anti-cancer potential against several cancers such as prostate, colon, breast, lung, and oral. SFN exerts its anti-cancer effects by inducing apoptosis, cell-cycle arrest, modulation of oncogenic signaling pathways, and the inhibition of angiogenesis. It acts by Nrf2 activation and HDAC inhibition [122].

#### 2.3.2. Diindolylmethane (DIM)

DIM is naturally presented as a glucosinolate conjugate in cruciferous vegetables and is released upon hydrolysis when the plant is damaged either by cutting or chewing. Kandala et al. showed the anti-cancer efficacy of DIM against ovarian cancer; wherein the anti-proliferative effect against ovarian cancer cells was observed due to induction of apoptosis and cell-cycle arrest in the G2/M phase [123]. They observed that DIM increased the phosphorylation of H2A.X and activated checkpoint kinase 2 (Chk2) [123] and downregulated the phosphorylated EGFR [124], MEK, and ERK. DIM also suppressed p-STAT3 [125], VEGF, and HIF-1α [125]. Decreases in VEGF and HIF-1α demonstrated that DIM significantly reduced cell-invasion and angiogenesis. The results were corroborated by the tumor inhibitory effects of orally administered DIM on the SKOV3 ovarian tumor xenografts in athymic nude mice [125]. DIM induced macroautophagy in ovarian cancer cells and activated AMPK, leading to apoptosis [126]. Anoikis is a mode of cell death that occurs after the cell detaches from the extracellular matrix (ECM). Cancer cells are resistant to anoikis, thus leading to enhanced cell proliferation and invasion [127]. Kandala et al. found that DIM reduced anoikis resistance through the downregulation of Gli1. The results were confirmed in vivo using Gli1 knockout cells in mice [128]. DIM has also been effective against breast cancer cells. Ganesan et al., out of the different derivatives of DIM, showed that DIM-1 and DIM-4 were the most potent variants. These compounds were able to inhibit cell migration and the activity of MMP-2 and MMP-9, indicating that DIM can prevent cancer metastasis. With an increase in the expression of cleaved PARP, cleaved caspase-3, and Bax and a decrease in the expression of Bcl-2, apoptosis was proposed to be the mode of cell death. The DIMs blocked the EGF receptor and thereby inhibited the Ras-mediated Akt/PI3K/mTOR signaling axis [129]. Munakarmi et al. found that DIM inhibited hepatocellular carcinoma (HCC) cell growth by inducing the caspase-dependent apoptotic pathway. DIM suppressed the epithelial–mesenchymal transition (EMT) by targeting the ER-stress and unfolded protein response (UPR) [130]. Several studies have also shown DIM to be effective against prostate cancer. Wang et al. found that DIM inhibits the LPS-mediated induction of IL1ß mRNA and protein in undifferentiated THP-1 monocytes. However, this was not the case in differentiated THP-1 macrophages. Also, knockdown studies showed that this effect was regulated by the aryl hydrocarbon (AHR) pathway. DIM inhibited CD84 mRNA but not the protein. Thus, it can be said that DIM treatment leads to crosstalk between AHR and the inflammation-mediated pathway in monocytes, resulting in modulation of the tumor microenvironment. However, this effect was not observed in macrophages. Also, the crosstalk was independent of the CD84-mediated pathways [131]. DIM was observed to exert its anticancer effects via inhibition of the PI3K/Akt/mTOR signaling pathway and the aryl hydrocarbon receptor pathway [132].

### 2.4. Cucurbitaceous Foods

Some vegetables and fruits belong to the Cucurbitaceae family. They are also called the gourd family, and their members are called the cucurbits. They include cucumber, melon, watermelon, pumpkin, gourd, squash, etc.

Cucurbits contain a triterpenoid steroid, cucurbitacin B (CuB), which has been shown to exhibit anti-tumor effects against several cancers. Gupta et al. showed the anti-proliferative effects of CuB against breast cancer cells at low concentrations, ranging from 18 to 50 nM, and acting via the downregulation of HER2 and integrin signaling. Several integrins (ITG) such as ITGA6, ITGB1, ITGB3, and ITGB4 have different roles. ITGA6 and ITGB4, which are overexpressed in breast cancer, were downregulated, whereas ITGB1 and ITGB3, which are responsible for causing integrin-mediated cell-death, were induced by CuB treatment. The anti-tumor efficacy was confirmed in the in vivo model, where Gupta et al. showed a significant inhibition of MDA-MB-231 and 4T-1 breast tumors injected orthotopically in BALB/c mice. Since 4T1 cells are the stage-IV representative of breast tumor, these findings bolster the significance of CuB being effective against breast cancer [133]. This research group also showed that cucurbitacin B helped suppress the metastasis of breast cancer cells to the brain [134].

Cucurbitacin B has also been effective in attenuating colorectal cancer, lung cancer, pancreatic cancer, breast cancer, neuroblastoma, and acute myeloid leukemia. It acts through the inhibition of STAT3 in all these cancers [133,135,136,137,138,139]. Several other mechanisms associated with anti-cancer effects of cucurbitacin B were the modulation of the EGFR pathway in colorectal cancer [139], the MAPK pathway in neuroblastoma [136], and inhibition of the CIP2A/PP2A/C-KIT signaling axis in myeloid leukemia [135].

Taken together, it can be inferred that cucurbitaceous foods have physiological effects against numerous cancers via the inhibition of oncogenic pathways, principally being the JAK/STAT axis.

### 2.5. Root Vegetables

Root vegetables are an edible portion of plants grown under the ground. Botanically they might be classified as root or non-root; however, collectively, they are referred to as root vegetables. Root vegetables with anti-cancer potential include ginger, garlic, beetroot, onion, carrots, turnips, sweet potato, and rutabagas. This review encompasses the molecular mechanisms underlying the anti-cancer effects of the phytochemicals in different root vegetables such as ginger, garlic, beetroot, and onion.

#### 2.5.1. Ginger (*Zingiber officinalis*)

Ginger is used as a taste enhancer in various types of foods and beverages and has shown anticancer activity in various cancer models. Ginger has demonstrated its efficacy in prostate cancer, wherein the whole extract of ginger was found to be more efficacious than the extract containing the phytoconstituents of ginger. This observation indicated the importance of the compound’s natural form, hinting that other natural components in ginger or any natural product may play essential roles in their overall activity [140]. Several phenolic compounds such as 6-gingerol, 6-shagol, zingerone, and 6-paradol have shown oncolytic effects in various experimental models [140,141,142,143,144,145,146,147,148,149,150,151]. Ginger extract inhibits breast tumor growth through the blockade of the G2/M phase. Researchers found that ginger caused cell death in breast cancer cells by different modes such as typical apoptosis, caspase-independent apoptosis, autophagy-dependent apoptosis, and autophagy [143,145,152,153]. Ray et al. observed that 6-shogaol, a phenol present in ginger, induced autophagic cell death in breast cancer cells along with the modulation of the Notch signaling pathway [143]. Lee et al. found that gingerol, another phenolic compound in ginger, inhibited the metastasis of MDA-MB-231 human breast cancer cells [154]. These findings were corroborated by Martin et al. with similar observation in vivo [155]. Another study showed a significant reduction in breast tumor growth in the orthotopic model of mice due to treatment with zerumbone, a cyclic sesquiterpene in ginger [152]. Seshadri et al. found that zingiberene, a constituent of ginger, inhibited the 7,12-dimethylbenz(a)anthracene-induced breast cancer growth in Sprague–Dawley rats [151]. Zerumbone and 6-shogaol treatment arrested the growth of prostate cancer cells (DU-145) in the G1 phase. This effect was attributed to the inhibition of STAT-3 and NF-κB signaling [156]. Similar inhibition of prostate cancer (PC3) tumor growth was observed in vivo by oral feeding of ginger extract [157]. Zerumbone treatment led to G2/M arrest in hepatocellular carcinoma cells. The underlying mechanism for this effect was the inhibition of PI3K/Akt/mTOR and the STAT3 signaling axis. The cells underwent cell-cycle arrest due to the shunting of glucose-6-phosphate in the pentose phosphate pathway [158].

Ginger and its phytoconstituents have also been effective against other cancers such as ovarian [158], colon [146], non-small lung [159], lung [159], gastric adenocarcinoma [160], melanoma [161], and cervical [162] by modulating NF-κB, p21, ERK1/2, p38, p53, Wnt/ß-catenin, and AMPK. Gingerols were also found to be effective in preventing emesis caused by chemotherapies [147]. Thus, the phytoconstituents in ginger not only help treat cancer but also prevent side effects due to chemo-drugs.

#### 2.5.2. Garlic (*Allium sativum*) and Onion (*Allium sepa*)

Onion and garlic are Allium vegetables belonging to the Amaryllidaceae family. They have been studied extensively for the treatment of cancer. Sulfur-containing compounds such as allicin, allylpropyl disulfide, diallyl sulfide (DAS), diallyl disulfide (DADS), and diallyl trisulfide (DATS) are responsible for the anti-cancer effects of garlic. Saud et al. demonstrated the efficacy of garlic against colitis-induced colon cancer through the modulation of the NF-κB pathway [163]. DADS suppresse breast cancer, liver cancer, and leukemia by inhibiting histone deacetylases. The results were confirmed via in vivo animal tumor models [164]. Studies have also observed that the organosulfur compounds in garlic induced phase-2 metabolizing enzymes to prevent cancer [165]. Hu et al. showed that DAS, DADS, and DATS, when administered to mice orally, induced the expression of α (mGSTA1-2, mGSTA3-3, mGSTA4-4), µ (mGSTM1-1), and π (mGSTP1-1) classes of GST enzymes in the lung, liver, and forestomach. However, mGSTP1-1 was most closely related to the inhibition of forestomach tumorigenesis induced by chemical carcinogens [166,167,168]. Thus, it can be said that organosulfur compounds in garlic act by modulating the phase-2 metabolizing enzymes, predominantly glutathione S-transferases (GST). The anticancer mechanisms of DADS and DATS involve ROS generation, downregulation of p-Akt and p-IGFR, upregulation of p-JNK/p-ERK along with G2/M cell-cycle arrest, induction of apoptosis, inhibition of histone deacetylases, and induction of phase-2 metabolic enzymes.

The efficacy of the bioactive compounds in onion has been observed against prostate, esophageal, colorectal, stomach, pharynx, larynx, renal, breast, ovary, and endometrial cancers [169]. Like garlic, onions also contain organosulfur compounds (OSCs), which exhibit anti-cancer properties. OSCs in onion control breast cancer by targeting heat shock protein HSP70, binding immunoglobulin protein (BiP), and stress inducible HSP70 [170]. Most of the tumor inhibition mechanisms of these OSCs remain similar to those of garlic compounds.

Taken together, the allium vegetables onion and garlic contain organosulfur compounds that are responsible for the antineoplastic effects in different cancer models.

#### 2.5.3. Beetroot (*Beta vulgaris* Subsp. *vulgaris*)

Betanin, a glyosidic food dye, is the principal component of beetroot responsible for its anti-cancer effect. Betanin induced apoptosis in cancer cells by activating the cleavage of caspase-3 followed by loss of the transmembrane potential of mitochondria [171]. Sreekanth et al. showed in vitro that betanin inhibited the proliferation of chronic myeloid leukemia human K562 cells, leading to apoptosis mediated by the release of cytochrome c from the mitochondria into the cytosol [172]. Betalain and betanine also have the potential to inhibit the growth of hepatocellular cancer (HepG2) cells [173]. Beetroot’s anti-cancer efficacy has been observed in the in vivo breast cancer models, wherein researchers showed a significant decrease in papillomas after treatment with 0.0025% betanin, a constituent of beetroot. The study also showed some promise against lung cancer in the in vivo model [10]. These findings provide a foundation to suggest the anti-cancer efficacy of beetroot.

### 2.6. Tropical Fruits

Tropical fruits are those grown in the hot and humid regions near the tropics of Cancer and Capricorn, covering the tropical regions of Asia, Africa, Central America, South America, the Caribbean, and Oceania. Of the several tropical fruits, Guava and Dragon fruit have shown strong anti-cancer potential. Their efficacy against several cancers and the mechanism of action are elaborated below.

#### 2.6.1. Guava (*Psidium guajava*)

The protective effects of guava extract have been shown against several cancers such as colorectal cancer, lung cancer, myeloid leukemia, myeloma, cervical cancer, squamous cell carcinoma, breast cancer, and gastric cancer [174,175,176,177]. Terpenoids in guava principally exhibit anti-cancer effects. A pharmacology network study by Jiang et al. showed that guava leaves were associated with several oncolytic mechanisms. In this study, Akt/PI3K, STAT3, and TP53 were the major players modulated by guava. Gene Ontology (GO) and Kyoto Encyclopedia of Genes and Genomes (KEGG) pathway analyses found 153 targets that were associated with small and non-small lung cancers affected by guava leaves [178]. Guava seeds contain a polysaccharide, guava seed polysaccharide fraction 3 (GSF3), which has been shown to inhibit breast cancer cell (MCF7) growth. Lin et al. proposed an increase in the Bax/Bcl-2 ratio and an increase in Fas mRNA expression as the probable mechanism [179]. Ryu et al. found that GHF, i.e., the hexane fraction of guava, exhibited anti-cancer potential against prostate cancer by modulating Akt/mTOR/S6K and MAPK pathways. The ERK/JNK/p38 signaling axis was also modulated in this study. In addition to GHF, the study also identified several other compounds, notably ß-eudesmol, α-copaene, α-patchoulene, ß-caryophyllene oxide (CPO), octadecane, and α-terpineol, for their anti-cancer efficacy [180]. Liu et al. found that guava extract inhibited the proliferation of several TNBC cells and induced apoptosis in them [181]. Rizzo et al. showed the efficacy of guava extracts in vivo against solid Ehrlich tumors at 50 mg/kg with no toxicity. Moreover, the guava treatment proved to be more efficacious than doxorubicin, the standard therapy of care [175]. The findings from the in vivo study corroborated the anti-cancer potential of guava. These findings also provide a promising foundation for deciphering the anti-cancer potential of guava in humans as well.

#### 2.6.2. Dragon Fruit (*Selenicereus undatus*)

Dragon fruit has shown anti-proliferative effects against prostate and colon cancer cells. Interestingly melanoma cells seemed relatively resistant to dragon fruit [182]. Dragon fruit exerts physiological effects due to the presence of antioxidant components such as betalains, steroids, and triterpenoids. Of these, betalains inhibit COX enzymes and lipid peroxidation. Betalain has been shown to inhibit the proliferation of breast (MCF-7), colon (HCT-116), stomach (AGS), glioblastoma (SF268), and lung (NCl-H460) cancer cells [183]. Betalain is also a component of beetroot. Although these findings look promising, more research is needed to corroborate the anti-cancer claims of dragon fruit and to decipher the detailed molecular mechanism.

### 2.7. Grass Family Members:

Lemongrass and wheatgrass are the food components belonging to the grass family, which exhibit anti-cancer properties.

#### 2.7.1. Lemongrass (*Cymbopogon citratus*)

Lemongrass is an essential component of different cuisines such as Thai, Vietnamese, Malaysian, Indian, and Southeast Asian foods, as well as in beverages. Lemongrass oil (LEO) consists of several terpenes and terpenoids such as citral, geraniol, α-bisabolol, geranyl acetate, and iso-intermedeol, which are responsible for its anticancer effects. LEO and its phytochemicals have been found to be effective against colon, liver, lung, cervical, oral, prostate, and brain (neuroblastoma) cancers. Overall, it was observed that LEO terpenes induce apoptosis by activating procaspase-3 and G2/M phase cell cycle arrest [184,185]. The double bond, conjugated with an aldehyde in the core of citral, serves as a potent casapsae-3 activator [185]. Citral also acts on several oncogenic pathways and leads to suppression of Src (Y416) and STAT3, as well as phosphorylation and inhibition of AMPK [186]. It also inhibits fatty acid synthase (FASN), leading to inhibition of the lipogenesis of prostate cancer cells [187]. Citral binds to microtubule affinity regulating kinase 4 (MARK4), an AMP-activated protein kinase, thus inhibiting its kinase activity [188]. Citral also modulates ERK1/2 and MAPK signaling cascades along with an increase in the phosphorylation of tumor suppressor p53 [189,190]. Geraniol, a terpene in lemongrass, possesses antiangiogenic effects, as confirmed by the suppression of VEGFR-2, leading to the reduction of Ki-67 and CD3-microvessels in vivo. It also increases ROS production, which helps in the killing of cancer cells [191]. Altogether, it can be said that lemongrass oil and its terpenes and terpenoids possess anti-cancer potential. However, human studies are lacking, and they will help in the translational aspects of the anticancer claims of lemongrass.

#### 2.7.2. Wheatgrass (*Thinopyrum intermedium*)

Wheatgrass is mainly consumed as a juice, and its powdered form is used in different cuisines. Although not much research has been done on wheatgrass for its anti-cancer potential, it has been used historically in different traditional forms of medicines, mainly the Ayurveda (Indian traditional medicine system). Studies have shown that the wheatgrass extract may help reduce metastasis by reducing the protein expression of VEGF, MMP-9, and COX-2 along with an increase in TIMP-2 in epithelial carcinoma (HEp2) cells. The anti-cancer effect was attributed to the inhibition of the Akt/PI3K pathway [192]. Sim et al. observed that the wheatgrass extract attenuates hypoxia induced factor (HIF1-α) and the mucins MUC5A, MUC5B, and MUC8 in A549 lung adenocarcinoma cells [193]. Wheatgrass juice has also been observed to be effective in reducing the vascular damage caused by chemotherapy in colon cancer and in increasing anti-inflammatory cytokine IL-10. These effects exhibit the protective actions of wheatgrass [194,195]. Overall, it seems that wheatgrass exerts antioxidant and anti-inflammatory effects. More robust in vitro and in vivo studies are needed to understand the underlying molecular mechanism of wheatgrass.

### 2.8. Caffeinated Plants (Tea (Camellia sinensis) and Coffee (Coffea arabica))

Tea and coffee are caffeinated plant products used as beverages worldwide. Caffeine, the principal component of tea and coffee, has promising anti-cancer effects. Although a few earlier studies indicated that coffee might help in cancer progression, research with better study designs later showed that coffee could potentially be useful against various cancers such as cancer of the head, neck, mouth, oral cavity, pharynx, throat [196], thyroid [197], liver [198], prostate [199], and endometrium [200]. Coffee has been shown to induce autophagy in vivo along with inhibiting the enzymatic activity of mammalian target rapamycin complex 1 (mTORC1) [201]. Kahweol, a diterpene in coffee, induces apoptosis in liver cancer cells acting through the Src/mTOR/STAT3 signaling pathway [202]. PT93, a phenolic derivative of caffeic acid, is present in coffee. It has been observed to be effective against glioblastoma (GBM). It acts by the inhibition of MMP-2 and MMP-9 in both in vitro and in vivo models. FLVM and FLVZ, the other two derivatives of caffeic acid, target IL17A, HIF-1α, and vascular endothelial growth factor (VEGF) by inhibiting angiogenesis [203]. Collectively, it was observed that caffeine arrests cancer cells in the G0/G1 phase through inhibition of Cdk4 and Cdk6. Caffeine and caffeic acid derivatives also act by inhibition of Akt, STAT3, p-ERK, p-FAK, and ROCK pathways [204] in liver cancer cells.

Epigallocatechin-3-gallate (EGCG), a catechin in green tea, is the main phytocompound that has shown tumor preventive properties against breast, kidney, colon, brain, leukemia, and prostate cancers [205,206]. EGCG inhibits tumor-associated macrophage (TAM) infiltration and M2 polarization [207]. In liver cancer cells, it activated AMPK and inactivated NF-κB [208,209]. EGCG inhibited c-Met signaling and upregulated p21 waf1, KIP1/p27, INK4a/p16, and INK4c/p18 expression in prostate cancer cells [210,211]. G2/M cell-cycle arrest, ROS generation, and downregulation of p-Akt and Wnt/ß-catenin pathway were a few common mechanisms by which EGCG exhibited its anticancer effects in different cancers.

### 2.9. Other Plant-Based Foods

**Figure 2:**
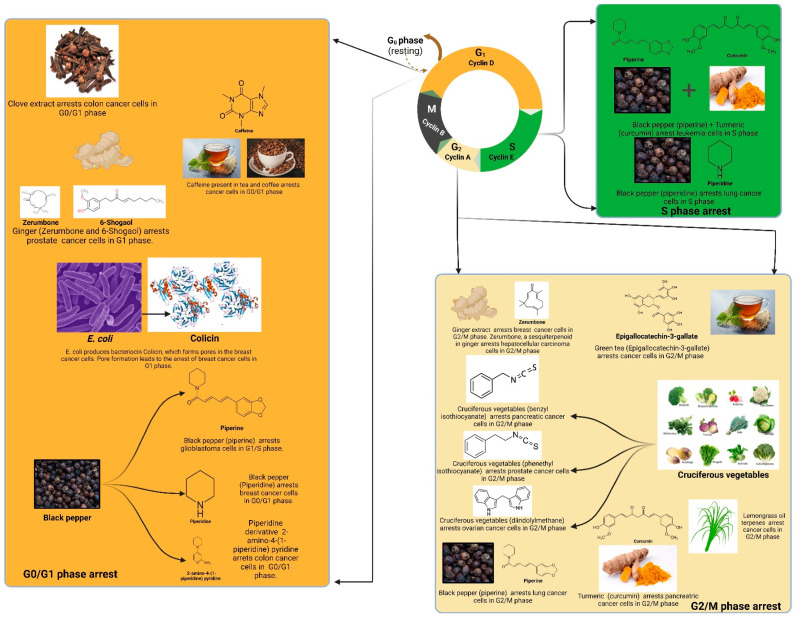
Various bioactive food components cause cell-cycle arrest at G0/G1, G2/M, and S phases in cancer cells. (Figure created with Biorender.com).

As seen in Figure 2, blueberries (*Vaccinium myrtillus*) contain pterostilbene, which alleviates breast cancer growth and its metastasis via suppression of the NF-κB/microRNA 448 circuit [212]. It also contains anthocyanins, which help against breast cancer [213]. Quinoa (*Chenopodium quinoa*) inhibits colon cancer by stimulating gastrointestinal digestion [214]. Caffeic acid in quinoa increases the G0/G1 population in HT29 (colon cancer) cells with the induction of apoptosis [215]. Avocado (*Persea americana*) has also been shown to induce the anti-cancer effects by inducing apoptosis, wherein Western blot analysis revealed an increase in cleaved caspase-3 and cleaved PARP levels. The concentration-dependent killing of cancer cells was also observed [216]. Pomegranate juice (*Punica granatum*) contains ellagic acid. The microbially generated metabolite (produced in human colonic microflora) urolithin is useful against colon, breast, and prostate cancers [217]. Citrus fruits have also been found to be useful against esophageal cancer [218] and breast cancer [219].

Table 1 summarizes the anti-cancer mechanisms of different plant-based food constituents along with the cell-lines that have been studied. 

## 3. Probiotics in Cancer

Probiotics have been shown to reduce cancer cell proliferation and induce apoptosis in vitro. *Lactobacillus paracasei* and *Lactobacillus rhamnosus* are Gram-positive bacteria that are used as probiotics. Orlando et al. showed the efficacy of these probiotics in mouse and human colon cancer cells, wherein an increase in apoptosis was observed [220]. Short chain fatty acids (SCFAs) are the metabolites of probiotics. It has been indicated that beneficial effects of probiotics are mediated via SCFAs [221]. SCFAs keep the gastric environment healthy by maintaining the appropriate acidity. They also prevent the formation of secondary bile acids and induce apoptosis in cancer cells [222]. Butyrate, a metabolite produced exhibits apoptotic effects in colorectal cancer cells. In order to enhance the effects of butyrate, probiotics are administered. SCFAs such as conjugated linoleic acid (CLA) induce the expression of apoptotic genes including caspase-3 and caspase-9 in colon cancer [223].

Additionally, it was found that SCFA-producing bacteria and other beneficial probiotics reduce the production of toxins and carcinogenic metabolites [224]. Compared to non-cancer colon tissues, colorectal cancer tissues have a less diverse microbial population [225]. Treatment with probiotics leads to a highly diverse microbial population, which might help reduce tumorigenesis or the further spread of cancer cells. Probiotics also reduce microbes from the *Fusibacter* genus, which are purported to be responsible for tumor initiation [226]. In general, probiotics play a crucial role in cancer prevention. The primary mechanisms of prevention include (1) downregulation of oncogenes, (2) cell cycle arrest, (3) inhibition of mutagens and carcinogens, (4) tumor suppressor gene activation, (5) induction of apoptotic and autophagic cell death, and (6) immune modulation for increased T cell infiltration [227]. Probiotics downregulate oncogenes such as MAPK, NF-κB, cyclin E, cyclin D, and β-catenin [228,229,230]. Downregulation of MAPK and NF-κB leads to apoptotic cell death [229]. For example, *Lactobacillus plantarum* (LPCLA) induced apoptosis in breast cancer cells by downregulating the NF-κB pathway, while other bacteria such as *Lactobacillus crispatus* and *L. rhamnosus* modulated the pro-oncogenic Wnt/β-catenin pathway in various cancer cell lines [231,232]. Another bacterial species, *Propionibacterium*, is known to induce apoptosis in colon cancer cells. The bacteria secrete propionate and acetate as the major cytotoxic components, which induce a three-phase apoptosis, mitochondrial alteration, caspase activation, followed by nuclear degradation. The two components caused mitochondrial membrane disruption followed by ROS generation and caspase activation [233]. Probiotics have also been observed to induce cell cycle arrest. For example, a bacteriocin called colicin produced by *E. coli* caused formation of pores on the plasma membrane of breast cancer cells while excluding normal human fibroblasts from this effect. Pore formation led to G1 phase cell cycle arrest [234]. Various studies have reported re-activation of tumor suppressor genes following probiotic treatment. For example, SCFAs produced by probiotics are known to cause epigenetic regulation and upregulation of tumor suppressor genes via metabiotic regulation of host specific physiological function. Metabiotics extracted from *L. rhamnosus* upregulated the expression of p53, a tumor suppressor gene in colon cancer [235]. Some probiotics also inhibit the metastatic spread of cancer cells. The secreted factors from *L. casei* and *L. rhamnosus* GG (LGG) downregulated matrix metalloproteinase-9 (MMP-9) and increased the level of tight junction protein ZO-1 in colon cancer [236,237].

An emerging role of probiotics in anti-cancer therapy is their immune modulatory effects. A study on the clinical responses to nivolumab and pembrolizumab concluded that fecal SCFA concentrations may be associated with anti-PDL1 efficacy. The findings of this study suggested that patients with high concentrations of fecal SCFAs, such as propionic acid, butyric acid, valeric acid, and acetic acid, had longer survival rates than their counterparts with no or less fecal SCFAs [238].

In a study by Shi et al., a combination of TGF-β receptor blockers and the *Escherichia coli* strain Nissle 1917 (EcN) led to a reduced immunosuppressive environment while increasing the infiltration of T cells and dendritic cell activation [239]. Another example of the microbiome modulating the tumor microenvironment (TME) is the presence of *Bacteroides* in melanoma. The activity of CTLA4 blockade treatment was enhanced in the presence of *Bacteroides thetaiotaomicron* or *Bacteroides fragilis.* In this study, mice that received antibiotic therapy did not responded to CTLA-4 blockade, but this was reversed after administrating *B. fragilis* through oral gavage [240]. In another study, renal cell carcinoma (RCC) patients who received probiotic supplements containing *Clostridium butyricum* showed a higher response to immune checkpoint blockade. The probiotic contained the *C. butyricum* strain CBM588, which was being widely used as an over-the-counter probiotic for anti-microbial-associated diarrhea [241]. In this study, patients received nivolumab and ipilimumab alone or with dual therapy along with probiotic CBM588. In both groups, progression-free survival was significantly higher than in the control arm. Also, as previously discussed, gut microbiota diversity was greater in patients who responded to the ICB therapy [242]. On the contrary, a study on immunotherapy in melanoma led by researchers from the National Cancer Institute (NCI) and the University of Texas M.D. Anderson Cancer Center found that probiotics did not improve the response to immunotherapy. The high fiber diet intake alone proved beneficial for potentiating the immune response without using any probiotic supplement. This is possibly due to the increase in healthy gut microbes following a high fiber diet [243]. This study leads to a question whether commercial probiotics for cancer immunotherapy provide an advantage. Further studies in larger cohorts will help in understanding whether the potentiating effect is cancer type specific.

It can be concluded that anti-cancer research with respect to probiotics is still in its infancy. Moreover, probiotic research is mainly focused on chemopreventive effects rather than therapeutic effects. Thus, more detailed mechanistic studies and clinical findings will help us substantiate the anti-cancer claims of probiotics.

**Figure 3:**
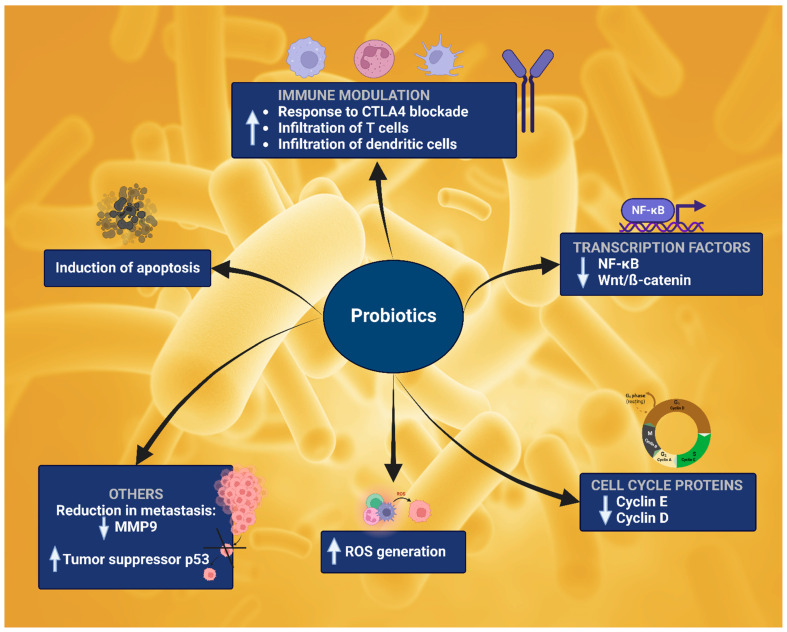
General scheme of molecular mechanisms by which probiotics exert anticancer effects. (Figure created with Biorender.com).

Different mechanisms of several probiotics along with the cell-lines that have been researched are mentioned in Table 2. Figure 3 summarizes the general scheme of mechanisms by which probiotics exert their anti-cancer effect.

## 4. Combination Strategies—The Therapeutic Opportunity

In order to exploit the food bioactive agents to the best of their potential, combination strategies are needed. The possible combinations can be (1) the combination of two or more food bioactive compounds, (2) the combination of food bioactive compounds with other physiologically active phytochemical/s, (3) the combination of food bioactive compounds along with chemotherapeutic agents, and (4) the combination of food bioactive compounds with immunotherapy.

### 4.1. Combination of Two or More Food Bioactive Compounds

Piperine and curcumin emulsosome was observed to be effective in treating colorectal cancer. The combination was more potent than the bioactive agent alone [244]. Li et al. observed that when leukemic cells were cotreated with piperine and curcumin, cells were arrested in the S phase. Moreover, the migration capability of these cells decreased significantly due to the cotreatment. The mode of cell death due to this combination was found to be both apoptosis and autophagy [29]. Khor et al. observed a significant reduction in PC-3 prostate tumor volume with the combination of PEITC and curcumin, as compared to PEITC or curcumin alone. This in vivo study exhibited a strong synergistic effect, where PEITC increased the efficacy of curcumin against prostate tumor [245].

Triphala is a well-known medicinal formulation consisting of a mixture of Indian gooseberry (*Emblica officinalis*), a fruit consumed extensively in the South Asian region, along with Haritaki (*Terminalia chebula*) and Vibhitaki (*Terminalia bellirica*), which are well-known medicinal natural herbs [246]. This polyherbal medicine, native to India, originates in Ayurveda, the Indian medicine system practiced for the last 3000 years. Triphala exhibits anti-inflammatory and antioxidant properties. It has been used to treat heart diseases, diabetes, premature ageing, and arthritis. Triphala’s anti-cancer effects have also been studied extensively along with these effects. Shi et al. studied the effects of Triphala against pancreatic cancer. This was the first-of-its-kind study deciphering the underlying molecular mechanism of the oncolytic effects of Triphala. Triphala was able to inhibit the proliferation of capan-2 pancreatic cancer cells. It induced apoptosis and enhanced ROS generation in capan-2 cells. In vivo, Triphala significantly inhibited capan-2 pancreatic tumor xenografts as compared to the control group. These effects were attributed to the activation of p53 and ERK. Thus, it can be said that Triphala exerts its pancreatic tumor suppressive effects by activating p53 and ERK [247].

### 4.2. Combination of Food Bioactive Compounds with Other Physiologically Active Phytochemical/s

Betacyanines (beetroot) when combined with vitexin-2-O-xyloside synergistically inhibited T24 urinary bladder cancer cells by inducing apoptosis. The synergistic combination leading to apoptosis was associated with the upregulation of the pro-apoptotic Bax, and downregulation of survivin, an inhibitor of apoptosis. The combination also downregulated ß-catenin expression, suggesting modulation of the ß-catenin signaling axis as the main mechanism [248].

### 4.3. Combination of Food Bioactive Compound along with Chemotherapeutic Agents

When coated with pectin, curcumin in combination with 5-fluorouracil was found to be useful against colorectal cancer [249]. In a study by Karthika et al., standard therapy 5-fluorouracil was combined with curcumin, and a synergistic effect was observed against the colon cancer cells (HCT116). The combination had the lowest IC_50_ when given in a ratio of 1:4, namely, one part 5-FU and four parts curcumin. This combination was coated with pectin, a citrus fruit constituent. Pectin is a polymer and is highly pH sensitive. Thus, it helped in the targeted delivery of the drug even when given orally. The study also found that titanium dioxide, part of many food items such as candies, was the root cause of cancer-induction in the colon [249]. It is important to know that certain food components can also cause cancer.

Piperlongumine and bortezomib have synergistic effects against renal cancer cells, with the downregulation of proto-oncoproteins [48]. In a study by Jeong et al., it was observed that piperine or TMZ alone could not inhibit the migration capability of GBM cells, as assessed by the wound healing assay. However, a combination of both led to the inhibition of migration. The combination also activated the JNK/p38 MAPK signaling axis [16]. Mitomycin C (MMC) is an anticancer antibiotic used to treat cervical cancer. However, due to multi-drug resistance, it was found that the tumors become resistant to this treatment. Han et al. studied the combined effects of piperine and MMC on cervical cancer cells and observed that the combination inhibited the proliferation of these cells. Moreover, piperine was able to decrease the resistance caused by MMC. The combination also suppressed p-STAT3 and NF-κB signaling. There is reported crosstalk between NF-κB and STAT3; thus, the mechanism of action of piperine and MMC combination could be through the reduced NF-κB/STAT3 signaling cross-talk. The combination treatment suppressed p65 expression in the nucleus and p-IκB expression in the cytoplasm, maintained a high Bax:Bcl-2 ratio and PARP and caspase-3, -8, and -9 activation, indicating apoptosis to be the mode of cell death [28,250]. A black pepper and doxorubicin combination increased cancer cell killing and decreased the genotoxicity of doxorubicin against ovarian cancer [251]. Piperine, when combined with MMC, inhibited cervical cancer growth [28].

Enzalutamide (ENZ) is an anti-cancer agent for the treatment of prostate cancer; however, cancer cells eventually become resistant to it. Tsao et al. evaluated the effects of the combination of 3,3′-diindolylmethane (DIM), a plant indole found in cruciferous vegetables, and ENZ against the prostate cancer cell-line 22Rv1. It was observed that DIM was able to produce anti-proliferative effects, even in ENZ-resistant cells. The combination led to the regulation of Wnt signaling, confirmed by the downregulation of ß-catenin, concomitant with the upregulation of GSK3ß and APC. The combination also decreased EMT along with the expression of androgen receptors [252].

Draz et al. observed that combining an autophagy inhibitor with DIM led to better tumor inhibition. The combination of DIM with the autophagy inhibitor chloroquine (CQ) significantly decreased prostate tumor growth in mice. Also, CQ treatment led to increased sensitization of prostate cancer cells to DIM treatment. Thus, DIM and CQ acted synergistically to inhibit prostate cancer [253].

Phenethyl isothiocyanate (PEITC), combined with doxorubicin, exhibited anti-tumor effects against brain cancer, downregulating HER2 and STAT3 [111]. Cang et al. appreciated the synergistic effect of phenethyl isothiocyanate (PEITC) and paclitaxel against breast cancer cells. PEITC improved paclitaxel’s efficacy, and this combination enhanced apoptosis in breast cancer cells along with the hyperacetylation of iα-tubulin [254]. Mukherjee et al. found that the combination of PEITC and doxorubicin sensitized cervical cancer cells to doxorubicin. The combination acted through the modulation of protein kinase C and telomerase along with activation of caspase-3 and -8 [255].

## 5. Novel Strategies for Enhanced Delivery of Natural Food Bioactive Agents for Cancer Chemoprevention

Researchers have been trying to develop novel drug delivery systems to improve the site-specific targeting of the bioactive compounds along with their enhanced efficacy. The use of nanotechnology and thus nanoformulations has been extensively explored [256,257,258,259]. Below are a few examples of novel drug delivery methods to enhance the efficacy of phytocompounds.

Abadi et al. prepared a nanoemulsion of cloves essential oil and tested its efficacy against HT29 colon cancer and HFF skin cancer cells. The nanoemulsion significantly reduced the proliferation of both cell lines [260]. Nirmala et al. found that an oil-based nanoscale emulsion of cloves buds was effective against thyroid cancer cells (HTh-7) and induced apoptosis in these cells [82].

Piplartine, also known as piperlongumine, is an alkaloid and the main phytochemical in black pepper. It has poor solubility, leading to the lack of proper formulations. Fofaria et al. formulated a nanoemulsion of piplartine and found that formulated piplartine had a greater oral bioavailability (1.5-fold) with anti-proliferative action against melanoma cells (A375 and B16). This nanoformulation exhibited higher solubility and stability with a low polydispersity index. Oral administration of piplartine nanoemulsion significantly inhibited melanoma tumor growth in mice [261]. Thus, this study is an example of how physiologically active food components can be exploited for their anti-cancer use. Piperlongumine’s (PL) hydrogel formulation was applied on mouse brain, resulting in the inhibition of U251 and U87 GBM tumors, exhibiting the enhancing effects of a hydrogel [49].

Qhattal et al. prepared a formulation of benzyl isothiocyanate (BITC) in a nano-emulsion form with the aim of enhancing its solubility and dissolution. The nano-emulsion significantly increased the accumulation of BITC in the tumor cells and enhanced permeability. Thus, a modification in the formulation could help enhance the drug action on the desired site, and the anti-cancer potential of BITC could be exploited [262].

## 6. Discussion

Certain fruits and vegetables have bioactive compounds principally responsible for their anti-cancer effects through apoptotic pathways. The action of these phytochemicals is extrapolated to the modulation of several cell signaling cascades [263]. Immune modulation is also one of the mechanisms by which several chemotherapeutic agents act [264]; however, it has not been studied extensively in the case of natural products. An overall trend in research shows that natural products have been studied mostly for their chemopreventive or chemoprotective effects; however, immunomodulatory effects remain to be discovered. It can also be seen that most of these anti-cancer food constituents arrest the progression of cell-cycle at different phases as illustrated in Figure 2.

Food components and natural products amalgamate several complex chemical compounds. Thus, extrapolating the physiological effect to a particular phytochemical could be erroneous. Expecting an effect using a single phytochemical would not always be the best idea, as the physiological effect exerted could be the concerted effect of the combination of several phytochemicals. Thus, an overall anti-cancer effect observed in several epidemiological studies may not corroborate molecular studies using a single phytochemical.

Several phytochemicals pose challenges due to poor solubility, poor bioavailability, or bad taste or odor, posing a big hurdle not only for patient compatibility but also for the appropriate delivery of the drug to its target sites. Although several researchers are working on enhancing the delivery system, an appreciable amount of work still needs to be done to enhance the drug delivery, bioavailability, site specific targeting, and patient compatibility. A study by Fofaria et al. is one example where the otherwise unexplored piplartine from black-pepper was discovered for its anti-tumor efficacy by formulating a nanoemulsion [261]. The prodrug approach is also a great option to enhance the specificity of the phytocompound and to avoid off-target effects.

While several studies show the anti-cancer promise of various phytochemicals in vitro, the translational ability of these compounds in humans will remain a question until these claims are bolstered with in vivo studies followed by clinical trials. Several published studies infer that the anti-cancer potential of phytochemicals is solely based on their cytotoxic effect against cancer cells, claiming anti-cancer efficacy would be overarching. This review omits the food components whose anti-cancer claim has been made solely based on cytotoxicity assays. A meta-analysis would help to assess the anti-cancer efficacy of these phytochemicals, which could be holistically explained more in another systematic review. However, it would be challenging in the case of a narrative review like this, as the primary focus here is to decipher the molecular mechanisms by which these actions are elicited. With the current literature available, although we see many promising anti-cancer effects, the studies with null or negative results cannot be overlooked.

There are several molecular players that act as double-edged swords. JNK and MAPK are two such players. They change their roles depending on the cell-type and cell-death mechanism [265,266]. Thus, in this review, it can be seen that in some studies, the mechanism responsible for the anti-cancer effect was due to the activation of the JNK/p38 MAPK axis, while in other studies, it was through their inhibition.

It can be seen that the mode of cell death by most food bioactives in this review is apoptosis, with a few exceptions of autophagy and anoikis. Although this hints that natural food bioactives act through apoptosis, we cannot overlook the possibility of other less studied pathways of cell death. Other cell death mechanisms such as necroptosis, pyroptosis, and ferroptosis are currently being studied. There is a high chance that these pathways may play a significant role in the efficacy of anticancer effects of several phytochemicals and may have been missed by researchers.

It can be seen that these functional foods have been effective in a wide range of concentrations. Thus, it can be challenging with the available information to decide on a particular dose. However, as far as the doses are concerned, several clinical trials have been conducted on a “trial and error basis”. The bioavailability of phytochemicals is still a concern; therefore, novel delivery methods are in the development to tackle this problem. Nonetheless, significant work needs to be done in this field.

## 7. Conclusions and Future Perspectives

**Figure 4:**
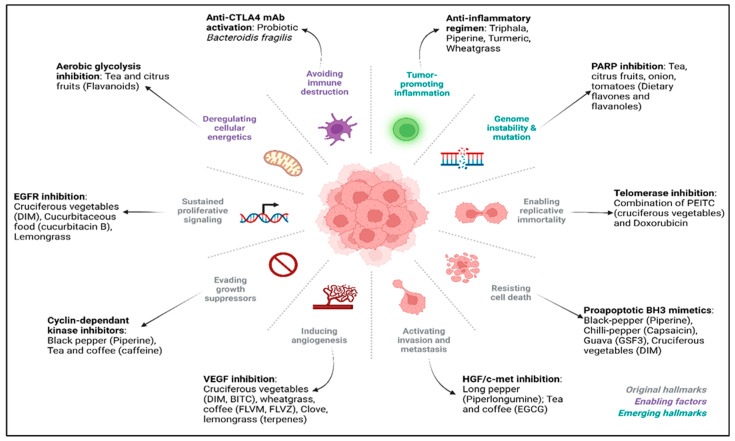
Hallmarks of cancer (original hallmarks, enabling factors, and emerging hallmarks) along with their corresponding therapeutic targets and various food constituents acting through it. (Figure created with Biorender.com).

Food bioactive agents exhibit promising anti-cancer effects and safe toxicity profiles, which can be attributed to the fact that they are food components. Most of the food constituents and functional foods act by targeting the hallmarks of cancer, as shown in Figure 4. A much deeper research design and more animal and human studies are needed to validate the anti-cancer claims of these agents. Natural herbs find their uniqueness in the fact that their effects are orchestrated due to the complex natural combination of bioactive chemicals they possess. In addition to studying the individual phytochemicals, deciphering their combination with other food bioactive agents or chemotherapeutics agents will help achieve synergistic effects. Enhanced drug delivery with specific targeting to the desired sites needs to be studied more to utilize this mother nature’s gift to the best of its potential in the treatment of cancer.

## Figures and Tables

**Table 1:** Mechanisms of different plant-based food bioactive agents used to treat cancer.

No.	Food	Bioactive Component	Cancer/Organ Model	Cell-Line	Mechanism of Action	References
1	Black pepper	Piperine	Breast cancer		**↓**pAKT, STAT3, p65, IκBα	[31]
			Ovarian cancer	A2780	**↓**JNK, p38 MAPK, STAT3	[13,14]
			Gastric cancer	TMK-1	**↓**IL-6	[14]
			Glioblastoma		**↓**CDK2-cyclin-E, CDK-4/6-cyclin D Jnk/p38 G1/S cell cycle arrest	[16]
			Lung cancer	A549	**↑**p53 G2/M cell cycle arrest and induction of intrinsic apoptosis	[17]
			Leukemia	HL60 and K-562	Induction of both intrinsic and extrinsic apoptotic pathway	[29,41]
			Colorectal cancer	HCT116, SW480	**↓**Wnt/β-catenin signaling	[43]
2	Black pepper	Piperidine	Breast cancer	MDA-MB-231 and MCF-7 cells	G0/G1 cell cycle arrest	[44]
		Piperidine (17a)	Prostate cancer	PC3	**↑**Bax **↓**Bcl-2 and XIAP	[24,45]
		Piperidine (2-amino-4-(1-piperidine)	Colon cancer	HT29 and DLD-1	G0/G1 cell cycle arrest **↓**FOXA2, vimentin	[46,47]
		Piperidine (CLEFMA)	Lung cancer	H441 cells	Induction of redox homeostasis Apoptosis induction S-phase cell cycle arrest **↓**COX2, cyclin D, NFκB, CD31	[90,92,94]
3	Long pepper	Piperlongumine	Renal cancer	PNX0010	**↓**c-met via ROS-dependent mechanism **↓**STAT3, NFκB, PI3K/Akt, and ERK/MAPK	[48]
		Piperlongumine	Glioblastoma	U87, U251	**↑**HTRPV2	[49]
		Piperlongumine	Cervical, pancreatic, and colorectal	HeLa, PANC1, and SW620	**↓**GSTP1	[50]
4	Chili pepper	Capsaicin	Pancreatic cancer	AsPC-1 and BxPC-3	Induction of ROS and apoptosis **↓**Bcl-2, survivin, β-catenin, JNK	[53]
		Capsaicin	Pancreatic cancer	AsPC-1 and BxPC-3	**↑**Inhibition of mitochondrial complex-1 and complex-3 followed by ROS generation	[54]
		Capsaicin	Bladder cancer		**↓**SIRT-1 deacetylase	[61]
5	Clove	Whole extract	Colon cancer	HT29	G0/G1 phase arrest **↓**E2F1, thymidylate synthase **↑**p21, WAF1/Cip1, γ-H2AX	[79]
		Whole extract	Breast cancer	MCF-7	**↑**ALDH1, caspase-3, and Bax **↓**CD44, CD24, and ALDH1	[80]
		Aqueous extract	Pancreatic and colon cancer	ASPC-1 and human colon HT-29	**↑**AMPK and ULK	[81]
		Nanoemulsion	Thyroid cancer	Hth-7, B-CPAP, BHT-101, and KTC-1 cell line	Apoptosis induction	[82]
6	Turmeric	Curcumin	Pancreatic cancer	BxPC-3	G2/M phase arrest **↑**pH2A.X, CHK1, p-ATM, **↓**Cyclin B1 DNA damage effect is regulated via ER stress pathway	[83,84]
		Curcumin	Oral squamous carcinoma	HSC-3 cells	Akt/PI3K/mTOR **↑**miR-199a	[86]
		Curcumin	Glioblastoma		**↓**MMP, NF-κB, STAT3, Akt	[87]
7	Cruciferous vegetables	Benzyl isothiocyanate	Pancreatic cancer	Capan-2	**↑**DNA damage G2/M phase arrest and apoptosis induction	[106]
		Benzyl isothiocyanate	Pancreatic cancer	BxPC3, MiaPaCa2, and Panc-1	**↓**STAT-3, PI3K/AKT/FOXO	[104,107]
		Benzyl isothiocyanate	Colon cancer	HT29	**↓**Urokinase-type plasminogen activator (uPA) protein kinase C (PKC), MAPK signaling pathway, and MMP-2/9 pathway	[102]
		Phenethyl isothiocyanate (PEITC)	Breast cancer	MDA-MB-231-BR (BR-brain seeking)	**↓**HER2	[110]
		PEITC	Breast cancer	MDA-MB-231 (in vivo xenografts in artificial immune environment)	**↓**MDSC cells	[112]
		PEITC	Prostate cancer	LNCap cells	Reactivation of tumor suppressor gene RASSF1A and apoptosis induction Cell cycle arrest and impact on global CpG epigenome	[113,114]
		PEITC	Chronic lymphocytic leukemia	Primary leukemia cells deficient in p53.	ROS generation via glutathione depletion	[115]
		Diindolylmethane (DIM)	Ovarian cancer	SKOV-3, TOV-21G, and OVCAR-3	**↑**p-H2A.X, Chk2 **↓**p-EGFR, MEK, ERK, p-STAT3, VEGF, and HIF-1α Induction of macroautophagy with AMPK activation	[123,124,126]
		DIM	Ovarian cancer	A2780 and OVCAR-429 cells	**↓**Gli-1	[128]
		DIM	Breast cancer	MDA-MB-231	**↓**MMP-2, MMP-9, and Akt/PI3K/mTOR signaling axis, EGFR	[129]
		DIM	Hepatocellular carcinoma	Hep3B and HuhCell	Suppression of EMT via ER stress induction	[130]
		Sulphoraphane	Prostate, colon, breast, lung, and oral		**↑**Nrf2 activation **↓**HDAC Apoptosis induction, cell cycle arrest and inhibition of angiogenesis.	[122]
8	Cucurbitaceous food	Cucurbitacin B (CuB)	Breast cancer		**↓**HER2 and ITGA and ITGA4 **↑**ITGB1 and ITGB3	[133]
		CuB	Colorectal cancer, lung cancer, pancreatic cancer, breast cancer, neuroblastoma, and acute myeloid leukemia		Inhibition of STAT3 signaling	[133,135]
		CuB	Colorectal cancer	HT-29 and HCT-116 cell lines	Inhibition of EGFR and JAK/STAT pathway	[139]
		CuB	Neuroblastoma	SHSY5Y cells	Inhibition of MAPK pathway	[136]
		CuB	Myeloid leukemia	Kasumi-1, acute promyelocytic leukemia (HL60), acute myelomonocytic leukemia (U937), chronic myelogenous leukemia (K562), Burkitt’s lymphoma (Raji) and T cell acute lymphoblastic leukemia (Molt-4)	CIP2A/PP2A/C-KIT signaling axis	[135]
9	Ginger	6-Shogaol	Breast cancer	MCF-7 and MDA-MB-231	Autophagic cell death and modulation of Notch signaling	[143]
		Gingerol, zerumbone, zingiberene	Breast cancer	MCF-7 and MDA-MB-231 Human brain seeking (MDA-MB-231BrM)	Metastatic inhibition and cell cycle arrest	[152,154,155]
		Zerumbone and 6-shogaol	Prostate cancer	DU-145	Cell cycle arrest in G1 phase **↓**STAT-3 and NF-κB	[156]
		Zerumbone	Hepatocellular carcinoma	HepG2, SNU-182, Hep3B, SNU-449, Sk-Hep-1, and Huh-7 cells	G2/M arrest **↓**PI3K/Akt/mTOR and STAT3 signaling axis	[158]
10	Garlic	Diallyl disulfide (DADS)	Colon cancer	SW480 cells	**↓**NF-κB	[163]
		Diallyl sulfide (DAS), DADS, and diallyl trisulfide (DATS)	Lung, liver, and forestomach		**↑**GST enzymes (α (mGSTA1-2, mGSTA3-3, mGSTA4-4), µ (mGSTM1-1), and π (mGSTP1-1))	[166,167,168]
11	Onion	Organosulfur compounds	Breast cancer		**↑**Heat shock proteins (HSP70) and binding immunoglobulin protein (BiP).	[170]
12	Beetroot	Betanin	Chronic myeloid leukemia	K562 cells	Induction of apoptosis by cytochrome c release from mitochondria	[172]
		Betalain and betanine	Hepatocellular cancer	HepG2 cells	Free radical scavenging activity	[173]
13	Guava	Guava seed polysaccharide fraction 3 (GSF3)	Breast cancer	MCF-7 cells	Apoptosis induction by increasing Bax/Bcl-2 ratio	[179]
		Guava leaf hexane fraction (GHF), ß-eudesmol, α-copaene, α-patchoulene, ß-caryophyllene oxide (CPO), octadecane, α-terpineol	Prostate cancer	PC-3 and LNCaP cells	**↓**Akt/mTOR/S6K and MAPK signaling	[180]
		Total extracts and smaller molecular weight (<30 kDa) extracts from guava fruit	Breast cancer	MDA-MB-231 and MDA-MB-468 cells	Apoptotic and necrotic cell death induction	[181]
14	Dragon fruit	Betalain	Breast cancer, colon cancer, stomach, glioblastoma, and lung cancer	MCF-7, HCT-116, AGS, SF268, and NCI-H460 cells	Inhibition of lipid peroxidation and COX enzymes	[183]
15	Lemongrass	Lemongrass oil terpenes	Lung cancer	A549, NCI-H1975, NCI-H1650, and NCI-H1299	**↑**Caspase-3 G2/M cell cycle arrest	[184,185]
		Citral	Small-cell lung cancer (SCLC)	LU135 SCLC cell line	Inhibition of Src/STAT3 activity	[186]
		Citral	Prostate cancer	PC-3 and PC-3M (metastatic)	Suppression of lipogenesis through inhibition of p-AMPK and downregulation of sterol regulatory element-binding protein (SREBP1), 3-hydroxy-3-methylglutaryl-coenzyme A reductase (HMGR), fatty acid synthase (FASN), and acetyl coA carboxylase (ACC)	[187]
		Citral	Colon cancer	HT29 and HCT-116	**↑**p53 and apoptosis induction via ROS generation	[190]
		Geraniol	Endometrial adenocarcinoma	Ishikawa cells	**↑**Bax, caspase3, caspase-8, cytochrome C, and Fas genes	[191]
16	Wheatgrass	Methanol extract of wheatgrass (MEWG)	Epithelial carcinoma	HEp2	**↓**VEGF, MMP-9, and COX-2 along with inhibition of Akt/PI3K signaling.	[192]
			Lung adenocarcinoma	A549 cells	**↓**HIF-1α, MUC5A, MUC5B, and MUC8	[193]
17	Coffee	Kahweol	Hepatocellular Carcinoma	Hep3B, SNU182, and SNU423	Inhibition of Src/mTOR/STAT3 signaling axis	[202]
		PT93 FLVM and FLVZ	Glioblastoma	U87 MG and DBTRG MG	PT93 inhibits MMP-2 and MMP-9 expression FLVM and FLVZ target IL17A, HIF-1α, and VEGF	[203]
18	Green tea	Epigallocatechin-3-gallate (EGCG)	Hepatocellular carcinoma	HLE, HepG2, HuH-7, and PLC/PRF/5	Inactivation of AMPK and NF-κB	[208,209]
		EGCG	Prostate cancer	DU145 cells	**↓**Phosphorylation of c-Met, Akt, and Erk	[210]
19	Blueberries	Pterostilbene	Breast cancer	MCF-7 and MDA-MB-231	**↓**NFkB/microRNA 448 circuit	[212]
		Anthocyanin	Breast cancer	MCF-7 and MDA-MB-231	-	[213]
20	Quinoa	Caffeic acid	Colon cancer	HT29	**↑**Gastrointestinal digestion, apoptosis, and G0/G1 cell cycle arrest	[214,215]
21	Avocado	Chloroform extract of avocado	Hepatocellular, oral, prostate, and breast cancer		**↑**Cleaved caspase-3 and cleaved PARP leading to apoptosis	[216]
22	Pomegranate juice	Ellagic acid, urolithin A and urolithin B	Breast cancer	MCF-7	**↓**17 Beta estradiol	[217]
23	Citrus fruits	-	Esophageal and breast cancers	-	-	[218,219]
24	Tomatoes	Tomatidine	Gastric cancer	-	Regulation of ISG genes	[68]
		Lycopene	Prostate cancer	-	Modulation of AKT/EZH2/androgen receptor signaling pathway	[69]
		Whole powder	Prostate cancer	-	-	[70]
		Lycopene	Hepatocellular carcinoma	-	**↓** cyclin D1, HIF-1α, and PCNA	[71]

**Table 2:** Mechanisms of different probiotics used to treat cancer.

No.	Probiotic	Type of Cancer	Cell-Line	Mechanism	References
1.	*Lactobacillus paracasei* *Lactobacillus rhamnosus*	Colon and gastric cancer	DLD-1 (colon) and HGC-27 (gastric)	Induction of apoptosis	[220]
2.	*Lactobacillus plantarum (LPCLA)*	Breast cancer	MDA-MB-231	**↓**NF-κB pathway	[230]
3.	*Lactobacillus crispatus* and *L. rhamnosus*	Various cancer types	HeLa, MDA-MB-231, and HT-29	**↓**Wnt/β-catenin pathway	[232]
4.	*Propionibacterium*	Colon cancer	HT-29 and Caco-2	Induction of apoptosis by secreting propionate and acetate	[233]
5.	*Escherichia coli*	Breast cancer	MCF-7, MDA-MB-231	G1 phase cell cycle arrest and pore formation through production of a chemical called colicin	[234]
6.	*L. rhamnosus*	Colon cancer	Animal study	**↑**Tumor suppressor p53	[235]
7.	*Lacticaseibacillus casei* and *L. rhamnosus*	Colon cancer	HCT-116	Inhibit metastatic spread by downregulating MMP-9	[236,237]
8.	*Escherichia coli* strain Nissle 1917 (EcN)	Liver and breast cancer	4T1 and H22 cell lines	**↑**Infiltration of T cells and dendritic cells	[239]
9.	*Bacteroides fragilis*	Sarcoma, melanoma, and colon carcinoma	MCA205 (sarcoma), MC38 (colon), and Ret melanoma model	**↑**Response to CTLA -4 blockade	[240]

## Note

The authors would like to apologize if inadvertently the work of other contributors in the field is not cited.

## Appendix A

List of fruits and vegetables covered in this review. Peppers: black-pepper, long-pepper, and chili-pepper; nightshade vegetables: eggplant and tomato; spices: cloves and curcumin; cruciferous vegetables: broccoli, cabbage, cauliflower, kale, mustard, watercress, and horseradish; cucurbitaceous food: cucumber, melon, watermelon, pumpkin, gourd, and squash; root vegetables: ginger, onion, garlic, and beetroot; tropical fruits: guava and dragon fruit; grass family members: wheatgrass, and lemongrass; caffeinated beverages: tea and coffee; others: blueberry, quinoa, avocado, pomegranate, and citrus fruits.
